# Unforgettable film music: The role of emotion in episodic long-term memory for music

**DOI:** 10.1186/1471-2202-9-48

**Published:** 2008-05-28

**Authors:** Susann Eschrich, Thomas F Münte, Eckart O Altenmüller

**Affiliations:** 1 Institute of Music Physiology and Musicians' Medicine, University of Music and Drama Hannover, Hohenzollernstrasse 47, 30161 Hannover, Germany; 2 Institute for Psychology II, Neuropsychology, University of Magdeburg Otto-von-Guericke, Universitätsplatz, Building 24, 39106 Magdeburg, Germany

## Abstract

**Background:**

Specific pieces of music can elicit strong emotions in listeners and, possibly in connection with these emotions, can be remembered even years later. However, episodic memory for emotional music compared with less emotional music has not yet been examined. We investigated whether emotional music is remembered better than less emotional music. Also, we examined the influence of musical structure on memory performance.

**Results:**

Recognition of 40 musical excerpts was investigated as a function of arousal, valence, and emotional intensity ratings of the music. In the first session the participants judged valence and arousal of the musical pieces. One week later, participants listened to the 40 old and 40 new musical excerpts randomly interspersed and were asked to make an old/new decision as well as to indicate arousal and valence of the pieces. Musical pieces that were rated as very positive were recognized significantly better.

**Conclusion:**

Musical excerpts rated as very positive are remembered better. Valence seems to be an important modulator of episodic long-term memory for music. Evidently, strong emotions related to the musical experience facilitate memory formation and retrieval.

## Background

Music is omnipresent, and many people listen to music because of the emotional richness it adds to their lives [1]. Music can be used to experimentally induce emotional states [2-4] including peak experiences, such as "chills" and "shivers down the spine" [1,5-7]. Frequently, such music can be remembered even years later, possibly due to the strong emotions it first elicited. Since music unfolds over time [8], music recognition requires that incoming sounds be mapped onto a stored long-term representation which contains invariant properties of the piece. Indeed, listeners seem to retain much information about the music they know and are very accurate in reproducing familiar music [9-11]. While some evidence has been found in favour of a dedicated memory store for music [12,13], this is still under debate.

Emotional verbal and pictorial stimuli are remembered better than non-emotional ones [14-20]. This is probably due to the interaction of emotional and memory processes in limbic structures [21-23] and can be explained by the semantic associative network model of memory by Bower [24] which proposes that emotions are used as contextual information linked to the to-be-remembered item. The semantic associative network model assumes that emotions are represented in a network of nodes together with words, pictures or music. Stimulation of emotion nodes creates spreading activation that lowers the threshold of excitation of all associatively linked nodes and thus helps to retrieve an emotional item from memory. Similar evidence for an effect of emotions on memory for musical stimuli is still lacking.

To fill this gap, we conducted a study investigating the influence of emotional properties of musical pieces on subsequent recognition performance for these pieces in a second session a few days later. This study is a prelude to a brain imaging experiment. In a previous pilot study we used piano music by J.S. Bach as stimuli to test the effect of emotions on musical memory [25]. The stimuli yielded neither a sufficient recognition nor sufficient emotional contrasts. Therefore, in the present study we used symphonic film music as a stimulus with more emotional impact which we expected to be remembered better. In the present study we also changed the design of the study in some points (i.e., a 5-point-rating scale instead of a 7-point-rating-scale, only one week between sessions, and a higher number of stimuli) and used a larger number of participants. We changed to a 5-point-rating scale because the rougher scaling can be answered more clearly and easily by the participants and leads to more reliable answers.

In keeping with the literature and our previous study, we used a dimensional model for measuring emotions with the dimensions "valence" and "arousal" [26,27] and asked to rate the respective dimensions on a 5-point-rating scale which resulted in five categories for each dimension.

"Arousal" refers to the excitation level elicited by the music (ranging from very relaxing to very exciting). Previous studies have shown that emotionally arousing stimuli are elaborated more deeply and thus are remembered better [16,28-31]. We therefore hypothesized that highly arousing musical excerpts should be remembered better.

Finally, "valence" is understood here as the emotional value on a continuum from negative to positive (or unpleasant to pleasant) elicited by a musical stimulus. Some studies have shown better memory performance for stimuli with positive valence [31-34] others for negative valence [35-37]. Music elicits predominantly pleasant feelings [38,39], and emotion induction by music is strongest for happy and peaceful music [40]. We therefore restricted the stimuli to a valence range of neutral to strongly positive according to a preassessment, and hypothesized that the latter should be associated with better recognition memory.

In this experiment we focused on felt emotion. Therefore, participants were asked to rate arousal, valence, and emotional intensity elicited in them by the music and not to indicate emotions they detected in the music. Several recent studies have shown that feeling emotion and the judgement of expressed emotion are different parts of emotions and are evaluated differently in music listening [41-43].

Inspired by memory research using the levels of processing framework [44,45], we also manipulated the participants' tasks during encoding: An "emotion group" was asked to rate the musical pieces with regard to valence, intensity and arousal during the encoding phase (deep, semantic processing), while the second "time-estimation group" only performed a more superficial task (length estimation). We hypothesized that the "emotion group" would show a better recognition performance than the "time-estimation group".

## Results

### Overall Recognition Performance

**Figure 1:**
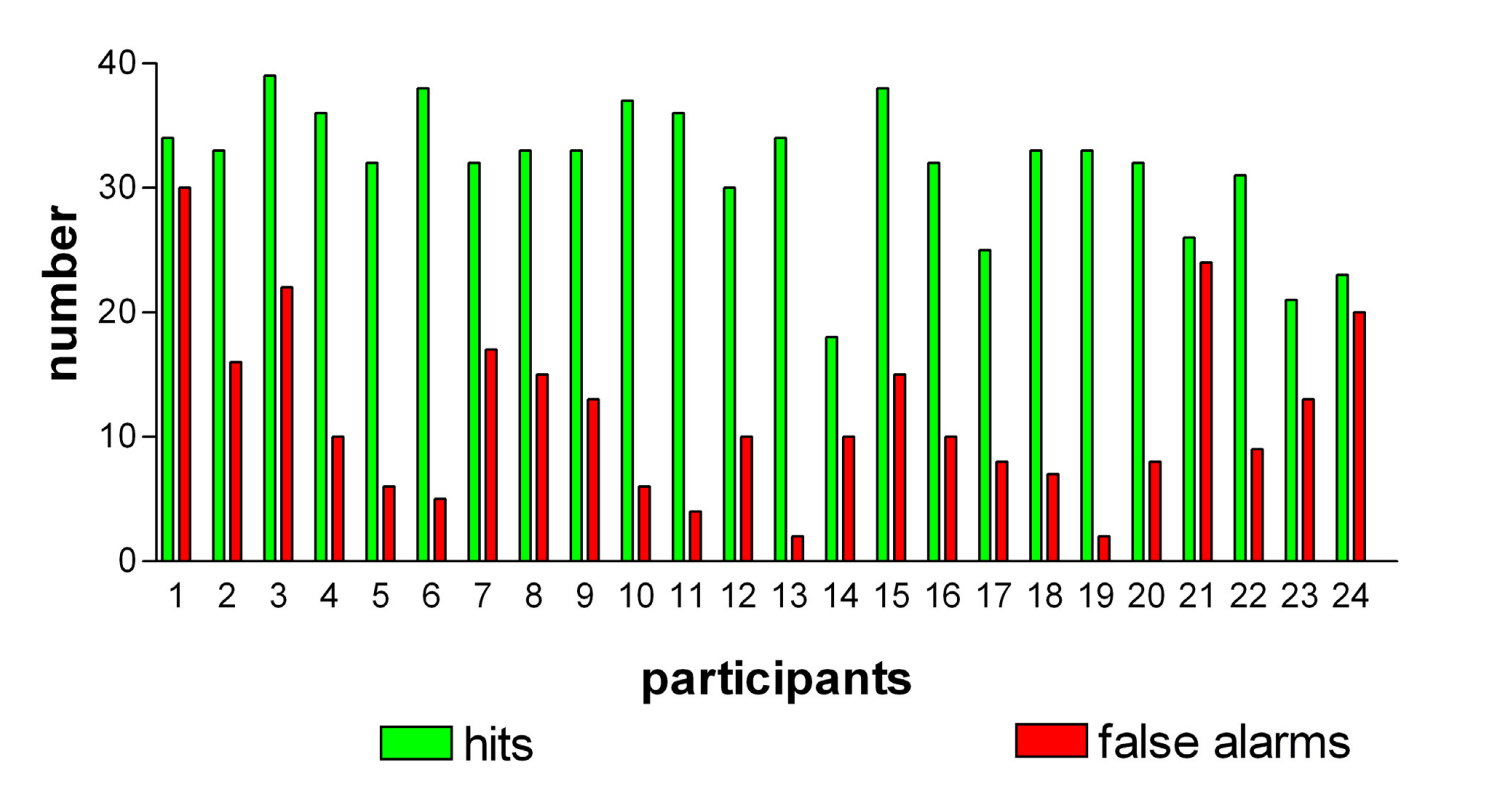
**Recognition performance per participant**. Number of correctly recognized targets (hits) and false alarms per participant. Total number of targets was 40.

The rates of correctly recognized targets (hits) and false alarms were calculated for each participant. The total number of targets was 40. The number of hits differed among participants from 21 to 39 with a median of 33 correct answers to targets and the rate of false alarms differed between 1 and 21 with a median of 7 incorrect answers to targets (N = 24) (Figure 1).

In addition, d' values were computed per participant. The d' values ranged from 0.13 to 2.77 with a mean of 1.51 (N = 24).

According to a reliability analysis the consistency of arousal ratings was very high (Cronbach's Alpha = 0.96, F = 25.59, p < 0.01, cases = 78). Valence rating (Cronbach's Alpha = 0.92, F = 12.34, p < 0.01, cases = 78) was also rather reliable.

Recognition performance (M-U-test of d' per participant: p = 0.71, N = 12) and emotional ratings (M-U-test arousal medians: p = 0.35; valence medians: p = 0.69, N = 12) did not differ significantly between the emotion group and the time estimation group.

Overall, the selected music pieces were not familiar to the participants before the experiment. Some of the participants had a feeling of familiarity in various music excerpts: three of the participants in eight, seven, and six pieces, respectively; two participants in five pieces; one knew four pieces; two in three pieces, and three in one piece. Except for three pieces which were familiar to two participants, the familiar pieces were all different for the different participants,. Even if the participants had indicated that the excerpt was familiar to them, they did not necessarily recognize this piece in the recognition session (only three participants correctly recognized all pieces indicated as familiar). Familiar pieces did also not receive higher valence ratings. We therefore decided to include all pieces of music in the analysis.

A reliability analysis was conducted to determine the consistency of the arousal and valence ratings for all stimuli and participants. For arousal ratings, reliability was very high (Cronbach's Alpha = 0.97, F = 28.7, p < 0.01, cases = 80). Valence ratings were also rather consistent (Cronbach's Alpha = 0.84, F = 6.25 p < 0.01, cases = 80). To test whether emotional ratings in the emotion group would change from the first session to the second session, a Wilcoxon test was calculated. For each excerpt of music, the median values of arousal, valence, and emotional intensity ratings of the emotion group members was compared between the two sessions. There was no significant difference between the arousal and valence ratings given in session 1 and those in session 3.

### Recognition memory and emotional rating

The following analysis was based on all 24 participants from the emotion and time estimation groups. The emotion ratings of the felt/induced emotion from the third session were used.

**Figure 2:**
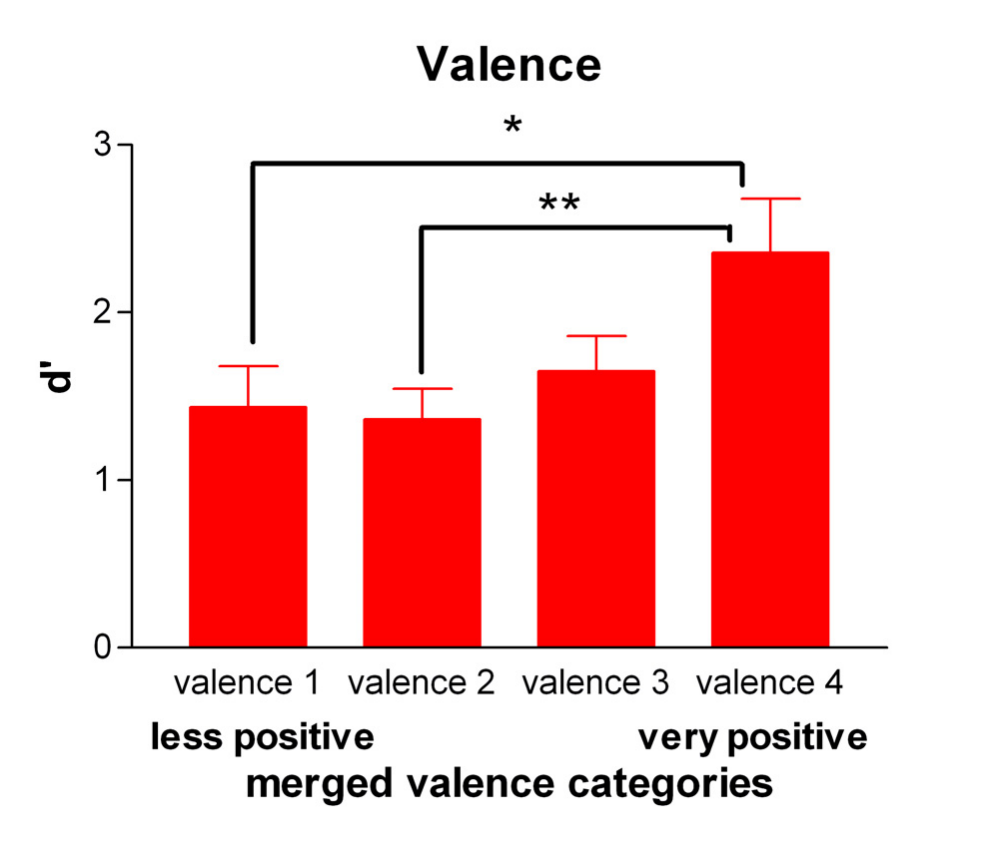
**Recognition memory and valence ratings**. Values of d' as a function of the valence category according to the individual ratings of each participant (2^nd ^session). The categories in the figure stand for the melted categories 1 + 2 = 1, 2 + 3 = 2, 3 + 4 = 3, 4 + 5 = 4. For details please see method section. *p < 0.05, **p < 0.01.

There was a significant effect of valence on recognition performance (category 1: d' = 1.4, categories 2 and 3: d' = 1.4 and 1.6, category 4: d' = 2.4; Friedman test, p = 0.002, N = 24; Figure 1). Dunn's multiple comparison tests revealed significant differences between category 1 vs. category 4 (p < 0.05) and category 2 vs. category 4 (p < 0.01) (Figure 2).

Recognition performance did not differ between the different arousal categories.

### Levels of processing: Emotion group versus time-estimation group

Even though a better recognition performance was expected in the emotion group, the overall d' values of both groups did not differ (t (18) = 0.6, p = 0.56, df = 18).

A 2 × 3 ANOVA (group, valence levels) with the dependent variable d' per valence category revealed no significant effects – neither a main effect for valence (F(1,18) = 1.67, p = 0.19, N = 20) nor for the group (F(1,18) = 0.39, p = 0.54, N = 20). There was no interaction between group and valence levels.

## Discussion

In this study, an incidental episodic recognition task was used to investigate whether music pieces which induce high arousal and very positive valence are remembered better by nonmusicians than are excerpts that rate as low arousing and emotionally neutral. We also examined the influence of depth of processing during the encoding phase on memory performance.

Despite the moderate recognition performance, clear-cut results concerning the relation between emotional ratings and musical long-term memory performance were obtained.

The results confirmed our hypothesis that music pieces which were rated more positively are positively related to the degree of recognition performance. Some studies in other domains showed a similar valence effect supporting this result [32,33].

However, some studies found negative valence [15,29,36] to improve memory. These results do not contradict the results of our study as it is perfectly possible that either negative or positive emotional stimuli could enhance memory performance. In this study we only tested the effect of music with neutral to positive valence on recognition performance. It could be that music rated as very negative would have similar enhancing effects on recognition performance as does very positive music.

Surprisingly, arousal ratings were not predictive for recognition performance. Thus the hypothesis that stimuli which induce high arousal are remembered better was not confirmed. Previous studies in other domains found that stimuli which induce higher arousal are remembered better independently of valence [14,15,19]. Other studies suggest that arousing stimuli attract attention [46] and are therefore processed more effectively and more elaborately [47,48]. Experience with other types of material, such as the IAPS pictures [49], indicates that strongest arousal is seen for very negative events. The use of neutral and positive material in the present study, therefore, might have precluded an arousal effect.

In summary, this study showed that ratings of valence are positively associated with better recall. However, it is possible that the ratings of emotionality in the second session may in part be based on the person's belief how memorable an excerpt of music was. The fact that the emotion ratings of the stimuli in the emotion group in the first session were very similar to those in the second session might speak against this assumption. Further studies are needed to ascertain on which aspect(s) of the stimulus the emotion ratings were based.

A further manipulation of the study concerned the task during the first (encoding) session: An emotional rating task, thought to give rise to deep elaborate processing, was contrasted with a time-estimation task, which was believed to lead to a more shallow processing of the musical pieces. Contrary to our hypothesis, as derived from the levels of processing framework, the recognition performance of the two groups did not differ. Because the processing level during encoding has profound influence on memory performance using other types of stimuli (e.g., words), this lack of an effect was surprising. A likely reason for this negative finding is that due to our experimental conditions (e.g., the fact that both groups had to answer a familiarity question after each piece during encoding), the processing level was not dissimilar enough for both groups. Our negative finding is not without precedence, however. None of the few studies using a level of processing manipulation in conjunction with musical material found an effect for (unfamiliar) music compared to verbal stimuli [50-52]. Thus, it has been assumed that musical memory differs from verbal memory [52].

Another possible explanation for the missing level of processing effect in music could be that music is not encoded in a fixed hierarchical manner as is language. The importance of different aspects of the music may change depending on the context of music processing or the setting. A further encoding task can therefore direct the attention of the listener to different aspects of the tune. Thus, the depth of encoding the features might not differ [52].

Of particular interest was the finding that the time-estimation group, despite not concentrating on the emotions during the first session, showed the same pattern of answers as the emotion group. The missing difference in recognition performance between both groups could also be explained by the small sample size of only 10 people per task group. However, both groups showed a similar distribution of correct answers in relation to the emotion ratings in the second session. Thus, the participants of the time-estimation group also implicitly processed the emotional content of the music and used it in the generation of a memory trace. In other words, emotion induced by the music is processed automatically and profoundly influences recognition.

## Conclusion

The results of this study indicate that emotional information modulates musical memory similar to the influence of emotional factors on memory in other domains.

Very positive valence ratings seem to be associated with better memory performance of music in a recognition task. Arousal ratings were not significantly related to recognition performance. This contrasts with the majority of memory studies in other domains which found arousal to be the most important variable in recognition memory of emotional events [14,15,19]. The level of processing manipulation in conjunction with the incidental memory task confirms that emotional information in music is processed automatically and implicitly. The neural underpinnings of this emotional modulation of musical memory are currently under study using an event-related fMRI design.

## Methods

### Participants

The protocol for the experiment was approved by the local ethics committee. Twenty-four nonmusicians (twelve women) gave informed consent to participate in the study for a small monetary compensation. They were undergraduate and graduate students of the University of Hanover with normal hearing abilities. The mean age was 25.5 years (range = 19 to 44 years). Only three of the 24 participants had learned to play an instrument or had sung in a choir for more than three years. However, all participants appreciated listening to music and said that music was important in their lives. We used nonmusicians to minimize the effect of musical structure on recognition, because nonmusicians are assumed to listen to music more on an emotional level and to not attend to musical structure in detail [50]. Additionally, nonmusicians do not have such a big repertoire of known music as do musicians, and we expected it to be easier to find music unfamiliar to them.

### Stimuli

A total of 80 excerpts of 20 to 30 s length (first session) or 10 s (second session) of symphonic film music by different composers [see Additional file 1] were selected from a larger pool by five musically trained raters. During this selection process, pieces with structural pop-outs, such as unexpected solo instruments or strange sounds or harmonies, were discarded, and preliminary arousal and valence ratings were obtained.

Using all pieces, we compared the median ratings of arousal and valence as well as the recognition of the pieces for short pieces with that for long ones with a cut-off at 27 s, and we compared the pieces with an extreme length (very short being up until 24 s and very long being over 27 s) in Mann-Whitney-U tests.

Longer pieces were not recognized better than shorter pieces (p = 0.09 and p = 0.08 (extreme lengths)), nor were they rated as more arousing (arousal: p = 0.9 and p = 0.7 (extreme lengths)) or more pleasant (valence: p = 0.6 and p = 0.3 (extreme lengths)).

All musical excerpts were edited to have the same dynamic range (46 dB to 66 dB). Two sets of 40 pieces were created with a comparable distribution of emotional and structural features according to the expert ratings on emotion and structure. Each of these sets was presented to half of the participants during the first session, while all stimuli were used during the second session. Concerning the five structural variables rated by the experts, the two sets of stimuli did not differ (complexity: p = 0.45; tempo: p = 0.75; loudness: p = 0.79; perceivability of melody: p = 0.61; number of motive repetitions: p = 0.92). After the experiment, the two sets of items were compared according to the participants' ratings of arousal and valence. Both item sets did not differ significantly with respect to any of these variables (Mann-Whitney-U-test for arousal, p = 0.9; M-U-test for valence, p = 0.1; M-U-test for intensity, p = 0.08). The proportion of the number of pieces per median of emotional rating category was the same for both sets.

### Procedure

During the experimental session, participants sat in a comfortable chair with a computer keyboard on their knees, and listened to the stimuli via closed headphones Beyerdynamic DT 770 PRO) and an USB soundcard (Audiophile, M-Audio). Questions and answer options appeared on the computer screen. Answers were logged by keyboard presses.

In both sessions, prior to the music rating, participants filled out the short mood questionnaire about their present arousal and valence (how "emotional" they felt at the moment) on a bipolar five-point rating-scale.

Participants then listened to excerpts of musical works of 20 s to 30 s (first session) or 10 s (second session). After the end of each excerpt, participants pressed a button to start the questions on the screen. Responses were not timed. After the last question, there was a break of 8 s before the new excerpt started. Excerpts were presented in randomized order in two blocks of 20 pieces in the first session and four blocks of 20 pieces in the second session. Blocks were separated by short breaks. The experiment was run on "Presentation 6.0".

For the first session, participants were divided into two groups of 10 persons: The emotion group was asked to rate the emotionality of each piece with regard to arousal and valence felt and perceived, while the time-estimation group was asked to estimate the length and general loudness of each stimulus. In addition to fulfilling the emotion or time-estimation task, participants were asked to indicate after each of the 40 stimuli whether the piece was familiar to them or not. During the encoding phase, participants were unaware of the subsequent recognition task in the second session.

Arousal, valence, and emotional intensity of each stimulus were rated on a five-point rating-scale (arousal: 1 = very relaxing/calming to 5 = very arousing; valence: 1 = little positive to 5 = very positive; intensity: 1 = no emotions at all to 5 = highly emotional). We used "less positive" (in German: wenig positiv) instead of "negative" because in a pretest none of the music pieces received a "negative" rating. Participants were asked to rate arousal and valence elicited in them by the piece of music (felt emotions). The estimation task comprised estimation of the total length of each excerpt and comparison to the length of the previous one. At the end of the first session participants filled out a questionnaire regarding demographic data, musical preferences and expertise as well as listening attitudes and experiences.

In order to facilitate memory consolidation, a second session, one day later, was introduced in which all participants listened to their respective target pieces again without any task. In the third session, on the third day, both groups listened to the 40 old stimuli from the last session randomly inter-mixed with 40 new pieces. All participants had to make an old/new decision followed by the emotion task.

### Data analysis

Values of d' were computed for each emotion category separately according to the individual valence and arousal ratings of each participant. We subtracted the z-corrected portion of wrong and guessed answers to distractors (false alarms) from the z-corrected portion of correctly recognized and guessed targets (hits). Only categories 2 to 4 were included because the number of pieces in the extreme categories was very small. For valence, five participants had no pieces in category 1, and seven pieces in category 5, seven participants (category 1) and four participants (category 5) had one or two pieces in these extreme categories. For arousal, two participants had no pieces in category 1, and five pieces in category 5, seven participants (category 1) and five participants (category 5) had one or two pieces in these extreme categories.

The d' values per category were compared using Friedman tests and a Dunn's multiple comparison test as post hoc test.

An ANOVA with a 2 × 3 design (group, valence) was used to compare the answer behaviour of the emotion group and time estimation group. Possible arousal, valence, and emotional intensity rating differences for the second rating between the two groups were tested with a Mann-Whitney-U test for each piece of music separately.

The consistency (Cronbach's Alpha) of arousal and valence ratings for each musical excerpt was examined with a reliability analysis.

## Authors' contributions

SE, EOA and TFM co-designed the study. SE performed and analysed the experiments, and wrote the first draft of the manuscript. EOA and SE selected the musical pieces. EOA and TFM wrote further drafts of the manuscript. All authors read and approved the final manuscript.

## Supplementary Material

Additional file 1 References of the music pieces used in the experiment. The reference list provided represents the music pieces from which excerpts were used in the experiment. Click here for file
