# Supplementary material for: Unforgettable film music: The role of emotion in episodic long-term memory for music
Source: BMC Neurosci. 2008 May 28;9:48. doi: 10.1186/1471-2202-9-48 (PMC2430709; doi:10.1186/1471-2202-9-48)
Supplement: Additional file 1 — References of the music pieces used in the experiment. The reference list provided represents the music pieces from which excerpts were used in the experiment. [file 1471-2202-9-48-S1.doc]

Medienverzeichnis

Addinsell, R. (1998). Warschauer Konzert [Recorded by P. Fowke (Klavier), P. Ó Duinn & RTE Concert Orchestra]. On *Warschauer Konzert.* *Große Klavierkonzerte aus Filmklassikern* [CD]. München: Naxos.

Arnold, D. (1996). Base Attack. On *Independence Day* [CD]. Los Angeles: BMG Classics.

Arnold, D. (2001). Main title. On *The Musketeer* [CD]. New York: DECCA Records.

Arnold, D. (2001). Fight inn. On *The Musketeer* [CD]. New York: DECCA Records.

Arnold, D. (2001). Prepare Duschamp for hell. On *The Musketeer* [CD]. New York: DECCA Records.

Arnold, D. (2001). Ride to Paris. On *The Musketeer* [CD]. New York: DECCA Records.

Arnold, D. (2001). The charge. On *The Musketeer* [CD]. New York: DECCA Records.

Auric, G. (1994). L’envolée. [Recorded by Adriano & Moscow Symphony Orchestra]. On *La Belle et la Bête* [CD]. München: MARCO POLO.

Badelt, K. (2003). Moonlight serenade. On *Pirates of the Caribbean* - *The Curse of the black pearl* [CD]. Burbank: Walt Disney Records.

Badelt, K. (2003). Underwater march. On *Pirates of the Caribbean* - *The Curse of the black pearl* [CD]. Burbank: Walt Disney Records.

Barry, J. (1990). Journey to Fort Sedgewick. On *Dances with Wolves* [CD]. Los Angeles: Epic Records.

Barry, J. (1990). The Buffalo hunt. On *Dances with Wolves* [CD]. Los Angeles: Epic Records.

Barry, J. (1990). The death of Cisco. On *Dances with Wolves* [CD]. Los Angeles: Epic Records.

Beltrami, M. (2004). Oct. 7^th^ 1944. On *Hell Boy* [CD]. Nürnberg: Varèse Sarabande Records.

Beltrami, M. (2004). Evil doers. On *Hell Boy* [CD]. Nürnberg: Varèse Sarabande Records.

Beltrami, M. (2004). Father’s funeral. On *Hell Boy* [CD]. Nürnberg: Varèse Sarabande Records.

Beltrami, M. (2004). Investigating Liz. On *Hell Boy* [CD]. Nürnberg: Varèse Sarabande Records.

Coulais, B. (2000). Helicopter. On *Die purpurnen Flüsse* [CD]. New York: Virgin Records.

Coulais, B. (2000). The Cable Car. On *Die purpurnen Flüsse* [CD]. New York: Virgin Records.

Farnon, R. (1992). The wind [Recorded by D. Towse & The Royal Philharmonic Orchestra]. On *Robert Farnon: Concert Works* [CD]. San Francisco: Reference Recordings.

Farnon, R. (1992). Lady Barabara [Recorded by D. Towse & The Royal Philharmonic Orchestra]. On *Robert Farnon: Concert Works* [CD]. San Francisco: Reference Recordings.

Fenton, G. (2004). Bounty Hunters [Recorded by G. Fenton & Berliner Philharmoniker]. On *Deep blue* [CD]. New York: Sony Classical.

Fenton, G. (2004). Coral riches [Recorded by G. Fenton & Berliner Philharmoniker]. On *Deep blue* [CD]. New York: Sony Classical.

Fenton, G. (2004). Flying emperors [Recorded by G. Fenton & Berliner Philharmoniker]. On *Deep blue* [CD]. New York: Sony Classical.

Fenton, G. (2004). Airwaves [Recorded by G. Fenton & Berliner Philharmoniker]. On *Deep blue* [CD]. New York: Sony Classical.

Fenton, G. (2004). Wolf Pack [Recorded by G. Fenton & Berliner Philharmoniker]. On *Deep blue* [CD]. New York: Sony Classical.

Fenton, G. (2004). Showtime [Recorded by G. Fenton & Berliner Philharmoniker]. On *Deep blue* [CD]. New York: Sony Classical.

Goldsmith, J. (1995). Lection Night/The Boys In The Prowl (Studs Lonigan). On *The Flim-Flam man – Studs Lonigan – Stagecoach* [CD]. Weiterstadt: Tsunami.

Goldsmith, J. (2001). Testing. On *Along came a spider* [CD]. Nürnberg: Varèse Sarabande Records.

Goldsmith, J. (2001). Megan’s abduction. On *Along came a spider* [CD]. Nürnberg: Varèse Sarabande Records.

Goldsmith, J. (2001). Megan overboard. On *Along came a spider* [CD]. Nürnberg: Varèse Sarabande Records.

Goldsmith, J. (2001). The ransom. On *Along came a spider* [CD]. Nürnberg: Varèse Sarabande Records.

Goldsmith, J. (2002). Final Flight. On *Star Trek Nemesis* [CD]. Nürnberg: Varèse Sarabande Records.

Herrmann, B. (1953). The Homecoming. On *Beneath the 12-Mile Reef* [CD]. Culver City: Film Score Monthly.

Herrman, B. (1991). Max. On *Cape Fear* [CD]. Universal City, CA: MCA Records.

Herrman, B. (1991). Love?. On *Cape Fear* [CD]. Universal City, CA: MCA Records.

Herrman, B. (1991). Strip search. On *Cape Fear* [CD]. Universal City, CA: MCA Records.

Herrman, B. (1991). Teddy bear wired. On *Cape Fear* [CD]. Universal City, CA: MCA Records.

Herrman, B. (1991). Kersek killed. On *Cape Fear* [CD]. Universal City, CA: MCA Records.

Herrman, B. (1991). The houseboat. On *Cape Fear* [CD]. Universal City, CA: MCA Records.

Horner, J. (1991). The Flying Circus. On *The Rocketeer* [CD]. Burbank: Hollywood Records.

Horner, J. (1991). Jenny. On *The Rocketeer* [CD]. Burbank: Hollywood Records.

Horner, J. (1991). Jenny’s Rescue. On *The Rocketeer* [CD]. Burbank: Hollywood Records.

Horner, J. (1993). Main title. On *A far off Place* [CD]. San Francisco: Intrada.

Horner, J. (1993). Attacked from the air. On *A far off Place* [CD]. San Francisco: Intrada.

Horner, J. (1993). Epilogue/End Credits. On *A far off Place* [CD]. San Francisco: Intrada.

Horner, J. (1994). Horror. On *The Pagemaster* [CD]. London: Fox Records.

Horner, J. (1994). A Narrow Escape. On *The Pagemaster* [CD]. London: Fox Records.

Horner, J. (1994). Towards the open sea.... On *The Pagemaster* [CD]. London: Fox Records.

Horner, J. (1994). The Flying Dragon. On *The Pagemaster* [CD]. London: Fox Records.

Horner, J. (1994). Swallowed alive. On *The Pagemaster* [CD]. London: Fox Records.

Kamen, M. (1998). Reunited/ Reincarnation/ When I was Young. On *What Dreams may come* [CD]. London: DECCA.

Korngold, E. W. (1991b). Baby Serenade op. 29 [Recorded by W. A. Albert & Nordwestdeutsche Philharmonie]. On *Erich Wolfgang Korngold – Orchestral Works Vol. 3* [CD]. Georgsmarienhütte: CPO.

Korngold, E. W. (1991b). Symphonic Serenade op. 39 [Recorded by W. A. Albert & Nordwestdeutsche Philharmonie]. On *Erich Wolfgang Korngold – Orchestral Works Vol. 3* [CD]. Georgsmarienhütte: CPO.

Korngold, E. W. (1991c). Theme and Variations op. 42 [Recorded by W. A. Albert & Nordwestdeutsche Philharmonie]. On *Erich Wolfgang Korngold – Orchestral Works Vol. 4* [CD]. Georgsmarienhütte: CPO.

Korngold, E. W. (1991a). Viel Lärmen um nichts op. 11 [Recorded by W. A. Albert & Nordwestdeutsche Philharmonie]. On *Erich Wolfgang Korngold – Orchestral Works Vol. 2* [CD]. Georgsmarienhütte: CPO.

Raksin, D. (1976). Forever Amber 1, 2, 5, 6, and 7 [Recorded by D. Raksin & New Philharmonia Orchestra]. On *Laura – Forever Amber – The Bad and the Beautiful* [CD]. New York City: BMG Classics.

Rozsa, Miklos (1959, 1996). Friendship. On *Ben Hur I* [CD]. Los Angeles: Rhino Records Inc.

Rozsa, Miklos (1959, 1996). Esther. On *Ben Hur I* [CD]. Los Angeles: Rhino Records Inc.

Rozsa, Miklos (1959, 1996). Love theme. On *Ben Hur I* [CD]. Los Angeles: Rhino Records Inc.

Rozsa, Miklos (1959, 1996). Rest. On *Ben Hur I* [CD]. Los Angeles: Rhino Records Inc.

Shearmur, E. (2004). The World of Tomorrow. On *Sky Captain and the World of Tomorrow* [CD]. New York: Sony Classical.

Shearmur, E. (2004). The zeppelin arrives. On *Sky Captain and the World of Tomorrow* [CD]. New York: Sony Classical.

Shearmur, E. (2004). The robot army. On *Sky Captain and the World of Tomorrow* [CD]. New York: Sony Classical.

Shearmur, E. (2004). The flying wing’s attack. On *Sky Captain and the World of Tomorrow* [CD]. New York: Sony Classical.

Shearmur, E. (2004). An Aquatic Escape. On *Sky Captain and the World of Tomorrow* [CD]. New York: Sony Classical.

Shearmur, E. (2004). Finding Frankie. On *Sky Captain and the World of Tomorrow* [CD]. New York: Sony Classical.

Williams, J. (1981). Main title: South America [Recorded by J. Williams & The London Symphony Orchestra]. On *Raiders of the Lost Ark* [CD]. London: Silvia Screen Records.

Williams, J. (1981). The Medaillon [Recorded by J. Williams & The London Symphony Orchestra]. On *Raiders of the Lost Ark* [CD]. London: Silvia Screen Records.

Williams, J. (1981). The Basket Game [Recorded by J. Williams & The London Symphony Orchestra]. On *Raiders of the Lost Ark* [CD]. London: Silvia Screen Records.

Williams, J. (1981). Marion’s Theme [Recorded by J. Williams & The London Symphony Orchestra]. On *Raiders of the Lost Ark* [CD]. London: Silvia Screen Records.

Williams, J. (1992a). High-Wire Stunts. On *Jurassic Park* [CD]. Universal City: MCA Records.

Williams, J. (1992b). Joe Sr.'s passsing/The duel scene. On *Far and Away* [CD]. Universal City: MCA Records.

Williams, J. (1992b). Burning the Manor house. On *Far and Away* [CD]. Universal City: MCA Records.

Williams, J. (1992b). Settling with Steven/The race to the river. On *Far and Away* [CD]. Universal City: MCA Records.

Williams, J. (1995). Der Kampf um Hollywood [Recorded by J. Williams & The Boston Pops Orchestra]. On *Williams on Williams. The Classic Spielberg Scores* [CD]. New York: Sony Classical.

Williams, J. (1995). Jim’s new life [Recorded by J. Williams & The Boston Pops Orchestra]. On *Williams on Williams*. *The Classic Spielberg Scores* [CD]. New York: Sony Classical.

Williams, J. (1997). Regaining a Son. On *Seven Years In Tibet* [CD]. Los Angeles: Mandalay Records.

Young, C. (1995). Main title. [Recorded by B. Gregor & The Czech Philharmonic Orchestra]. On *Copycat* [CD]. New York: Sony BMG.

Zimmer, H. (2003). Taken. On *The last Samurai* [CD]. New York: ELEKTRA.
